# Risk Factors for Foot Amputation in Patients Hospitalized for Diabetic Foot Infection

**DOI:** 10.1155/2016/8931508

**Published:** 2016-02-22

**Authors:** Maria Teresa Verrone Quilici, Fernando de Sá Del Fiol, Alexandre Eduardo Franzin Vieira, Maria Inês Toledo

**Affiliations:** ^1^Pontifícia Universidade Católica de São Paulo, Sorocaba, SP, Brazil; ^2^University of Sorocaba, Rodovia Raposo Tavares, Km 92,5, 18023-000 Sorocaba, SP, Brazil; ^3^Universidade de Brasília, Brasília, DF, Brazil

## Abstract

The aim of this study was to identify and quantify risk factors for amputation in diabetic patients hospitalized for foot infections. This cross-sectional study comprised 100 patients with diabetic infectious complications in the lower limbs. The variables investigated were related to diabetes, infection, and treatment compliance. Multiple Cox regression analysis was performed to identify the variables independently associated with the outcome of amputation. The most prevalent chronic complications were neuropathy and hypertension. Most patients presented with a neuroischemic foot (86%). The Morisky test showed that 72% were not compliant with diabetes treatment. Regarding patient outcome, 61% progressed to amputation, 14% to debridement, and 9% to revascularization. The results showed a 42% higher risk for progression to amputation in patients with previous use of antimicrobials. Also, the amputation risk was 26% higher for those less compliant with diabetes treatment. An increase of one point in the Wagner ulcer classification criteria corresponded to a 65% increase in the risk of amputation. Undergoing conservative, nonsurgical procedures prior to admission provided a 63% reduction in the risk of amputation. Knowledge of these factors is critical to enable multidisciplinary teams to develop treatment plans for these patients so as to prevent the need for amputation.

## 1. Introduction

Worldwide, the population with diabetes is currently estimated at 366 million and is expected to exceed half a billion by 2030 [1]. Foot ulcers are the principal cause of severe complications and hospitalization among patients with diabetes, substantially increasing the costs with this disease [2]. In the United States, the annual cost of foot ulcers is estimated at US$11 billion [3].

In Brazil, the population aged 30 years and over with type 2 diabetes is estimated at 6.5 million. Among these, roughly 323 000 cases of foot ulcers are reported annually, 97 000 of which require hospitalization [4].

Adding to the costs of managing infection, patients with diabetes are confronted with the risk of limb amputation, with rates 30 to 40 times higher than in individuals without the disease [2]. Studies have shown the incidence of diabetic foot to be on the order of 3% to 4%, accounting for roughly 11 million patients with this condition in 2014 [5, 6].

Peripheral neuropathy, ulceration, infection, and peripheral vascular disease are the principal factors for ulcer complications and loss of a lower limb in diabetic patients [7, 8]. Nonetheless, ambiguity remains as to which factors are most conducive to amputation outcomes and how strongly they affect these events [9]. Structured healthcare is one of the most effective approaches to reducing the indicators for diabetic foot amputation, and studies have shown that these can be reduced by as much as 75% [8].

Factors such as low socioeconomic status, smoking [10, 11], gender, renal impairment [12], ischemia, diabetic neuropathy [13], and high levels of glucose and triglycerides [14] have been reported as importantly associated with the risk of foot amputation.

This study evaluated the effect that clinical, biochemical, epidemiological, and patient-behavior-related predictors have on amputation outcomes in patients with diabetic foot. Knowledge of these factors and their influence on this outcome is critical to enable multidisciplinary teams to develop management and treatment plans for diabetic patients so as to prevent the need for foot amputation.

## 2. Material and Methods

This cross-sectional study comprised 100 patients with diabetic foot hospitalized at the Vascular Surgery Clinic of the Conjunto Hospitalar de Sorocaba, in Sorocaba county, São Paulo state, southeastern Brazil. Inclusion criteria were minimum age of 18 years, diagnosis of diabetes, presence of infected ulcers on a lower limb, and agreement to participate (expressed by signing a consent form). The project was approved by the Research Ethics Committee of the Universidade de Sorocaba (opinion 0028/10) and complied with the ethical standards laid down in the 1964 Declaration of Helsinki and its later amendments.

The patients responded to a structured questionnaire about their sociodemographic status, knowledge of the disease, previous antibiotic use, and compliance with diabetes treatment.

Data on the clinical characteristics and health status of patients were collected from medical records. The clinical and laboratory evaluations were performed at the Laboratory for Diabetes “Conjunto Hospitalar de Sorocaba.” Comorbidities had been evaluated by a group of specialists, based on consensus and guidelines [15–19]. These medical evaluations were available from the patients' records.

All foot ulcers were graded according to Wagner criteria [20]. Grade 1 ulcers are superficial, involving full skin thickness. Grade 2 ulcers are deeper, penetrating down to ligaments and joint capsule. Those of Grade 3 are deep lesions, with abscesses or osteomyelitis. Grade 4 ulcers exhibit localized gangrene. Grade 5 includes extensive gangrene, compromising more than two-thirds of foot.

Data analysis was based on debridement, revascularization, and amputation outcomes.

Compliance with outpatient treatment for diabetes was evaluated using the Morisky test [21], which consists of four simple questions. Do you ever forget to take your medication? Do you ever have problems remembering to take your medication? When you feel better, do you sometimes stop taking your medication? Sometimes, if you feel worse when you take you medication, do you stop taking it? Each negative answer is assigned one point. The higher the score, the more adherent the patient [21].

### 2.1. Statistical Analysis

Given the high prevalence of limb amputation, we estimated prevalence ratios and their respective confidence intervals (95% CI) for the univariate analysis of the relationships between variables and outcomes, using Shapiro-Wilk test, Student's *t*-test, or Mann-Whitney test. The variables with *p* values of less than 0.25 were selected for multivariate analysis using the Cox regression model with robust variance. The tests were performed at a significance level of 5%. All data were analyzed with Stata 11.0 statistical software (Stata Corp. LP, College Station, Texas, USA).

## 3. Results

Table 1 shows that age of the patients (*n* = 100; 32 women, 68 men) ranged from 31.9 to 89.7 years (median: 62 years), with 55% of patients older than 60. Most patients were male (68%), Caucasian (78%), poorly educated (69%), nonsmokers (81%), and alcoholics (84%) and had type 2 diabetes (99%). Of the total, 22% had been diabetic for less than five years, 24% from five to 10 years, 17% from 10 to 15 years, 16% from 15 and 20 years, and 21% for more than 20 years.

In most patients, diabetes was being monitored (79%). Most had attended annual medical appointments (73%) and, over the past year, had attended more than three appointments (67%) and tested for blood glucose levels (86%). Glucose levels at admission ranged from 4.10 to 28.7 mmol/L (mean: 12.43 ± 5.03 mmol/L).

The most frequent chronic complications were neuropathy (91%), hypertension (72%), vascular peripheral disease (63%), retinopathy (42%), dyslipidemia (41%), nephropathy (26%), coronary insufficiency (23%), and cerebrovascular insufficiency (16%). On admission, 75% of patients had Grade 4 ulcers, while 20% had Grade 3 and 5% had Grade 2 ulcers.

Less than half of the patients had undergone a prior conventional, nonsurgical procedure (debridement) (45%) or amputation (32%). For 74%, this was the first hospitalization for complications of diabetes. Most, however, had an ulcer of less than 2 cm (84%), gangrene (76%), and a neuroischemic diabetic foot (86%). Most patients showed signs of inflammation (89%) and had osteomyelitis (52%), which was also present with the high incidence of Grade 4 ulcers (75%).

Compliance with treatment was poor in 72% of patients (score 2 for 35 individuals, score 3 for 15, and score 4 for 15), while 27 were considered compliant (score 0 for 23 patients and score 1 for five).

### 3.1. Univariate Analysis

No statistically significant differences were observed in the prevalence of diabetic foot amputation with regard to gender, ethnicity, schooling, monthly income, alcohol consumption, or smoking.

No statistically significant differences in the prevalence of diabetic foot amputation were detected based on the occurrence of comorbidities. However, 75% of patients with two or three previous hospital admissions for chronic complications required foot amputation, whereas only 52.6% of those with one single admission experienced this outcome (*p* = 0.043; Table 2).

Table 3 shows that 78.6% of poor compliers (Morisky scores 0 or 1) had a foot amputated, whereas only 54.2% of compliant patients (scores 2–4) did so (*p* = 0.012).

Patients with a history of conservative procedures had a lower prevalence of amputation than those not subjected to this procedure (*p* < 0.001). However, previous amputation was unrelated to an amputation outcome (*p* = 0.255). Also, amputations were more frequent in patients with osteomyelitis than those lacking this condition (*p* < 0.001; Table 4).

### 3.2. Multivariate Analysis

To identify variables independently associated with progression to amputation, Cox multiple regressions (with robust variance) were performed on variables that showed *p* values lower than 0.25 on univariate analysis.

The association between ulcer grade (Wagner criteria) and treatment compliance score (Morisky test) was statistically significant (*p* = 0.014, chi-squared test). The prevalence of gangrene in patients with higher treatment compliance was 68.1%, rising to 92.8% in less compliant individuals (Morisky scores 0 or 1; Wagner Grade 4). Therefore, two models were found on multivariate analysis: one using the Morisky test (Table 5) and the other employing Wagner criteria (Table 6). Amputation outcomes proved independently associated with previous conservative procedures, previous use of antibiotics, and Morisky test scores or Wagner criteria (Tables 5 and 6).

The risk of foot amputation for patients who had received conservative treatment was 63% lower than for those with a previous amputation (*p* < 0.001; Table 5), while for individuals previously treated with antibiotics the risk of foot amputation was 42% higher than for patients not subjected to this drug therapy (*p* = 0.026).

Considering Wagner grades, the risk of foot amputation was 61% lower in individuals who had previously undergone conservative procedures than in those who had not (*p* < 0.001), Table 6. Among those previously treated with antibiotics, this risk was 36% higher than for those without antibiotic therapy (*p* = 0.042). Furthermore, for each unit increment in Wagner grade, there was a 65% increase in the risk of foot amputation in patients admitted with infectious complications in a lower limb (*p* = 0.018).

## 4. Discussion

In most subjects (81%), blood glucose levels ranged from 5.55 to 16.65 mmol/L. Glucose levels below 11.09 mmol/L at admission are associated with lower morbidity and mortality, and proper glycemic control is a critical factor for the infection eradication and ulcer healing. Chronic hyperglycemia is the most frequent etiological factor for complications of diabetes mellitus [22–25].

Neuropathy was reported in 91% of patients, coinciding with published data indicating a high prevalence of neuropathy in diabetic patients hospitalized for foot injuries [26]. Retinal impairment and nephropathy are the two most common microvascular complications, both of which were present in the study population (at 42% and 26%, resp.). In patients with diabetes, nephropathy is a marker for generalized vascular disease, and these patients are probably more susceptible to developing peripheral vascular disease [27]. Recent studies also suggest that the incidence of diabetic foot ulcers is more frequent in individuals with micro- and macroalbuminuria [28–30].

Patients who reported prior use of antibiotics had a 42% higher risk of major amputation than those not receiving antibiotic therapy. Similar results have been found in other studies [31, 32]. Previous prolonged use of antibiotics selects for resistant microorganisms, making treatment more difficult and increasing the risk of amputation.

The present data suggest an increased risk of amputation in patients less compliant with drug therapy. Adherence to the prescribed therapy has led to significant improvements in the health and quality of life of patients with diabetes [7, 33–37].

Compliance with medication is essential in chronic diabetes, improving control of disease progression and attenuating the severity of chronic complications. Reinforcement of guidelines on diabetes care and the importance of medication, both of which can increase treatment compliance, are facilitated when patients have more than three medical appointments per year.

In the present investigation, patients with a history of antibiotic use had an increased risk of progressing to amputation. Each unit increment in ulcer severity (measured in Wagner grades) increased the risk of amputation. Similar results were found in a Brazilian study that demonstrated a directly proportional relationship between Wagner grade and risk of limb amputation [38]. It is worth noting, however, that the Morisky test was originally developed for hypertension but has been used to evaluate drug treatment in patients with diabetes [39], a feature that may constitute a limitation of the present study. Another limitation is that information on previous use of antibiotics was self-reported rather than collected from medical records.

Noncompliance with pharmacological treatment of diabetes was associated with an increased risk of amputation. This risk was lower for patients who had undergone conservative treatment prior to admission.

Studies evaluating the extent of problems related to diabetic foot can provide elements for intervention policies and prevention programs—particularly in government-funded healthcare services—involving multidisciplinary teams specialized in diabetic foot care, ultimately ensuring improved treatment with more efficient use of resources.

## 5. Conclusion

The present findings highlight that antimicrobial therapy protocols for outpatients with diabetic foot need reviewing. Control of the disease before hospitalization can significantly reduce amputations in patients with diabetic foot.

Knowledge of these factors and their influence on amputation outcomes is critical to allow multidisciplinary teams to develop management and treatment protocols for patients with diabetes. The present findings show that limb amputation outcomes were strongly lowered by conservative treatment and compliance with diabetes drug therapy. Implemented in a preventive manner, these two measures can significantly reduce lower limb amputation in patients with diabetes.

## Figures and Tables

**Table 1:** Distribution of patients with diabetes by their sociodemographic characteristics and outcomes for diabetic foot amputation.

Characteristics	Amputation		PR (95% CI)	*p*
	No *n* (prevalence %)	Yes *n* (prevalence %)		
Gender				0.521
Male	25 (36.8)	43 (63.2)	1	
Female	14 (43.8)	18 (56.2)	0.89 (0.62–1.27)	
Caucasian				0.281
No	11 (50.0)	11 (50.0)	1	
Yes	28 (35.9)	50 (64.1)	1.28 (0.82–2.01)	
Schooling (years)				0.709
0 to 4	25 (36.2)	44 (63.8)	1	
5 to 8	11 (44.0)	14 (56.0)	0.88 (0.59–1.30)	
>8	3 (50.0)	3 (50.0)	0.78 (0.34–1.79)	
Total income (US$/month)				0.779
<900.00	34 (39.5)	52 (60.5)	1	
>901.00	5 (35.7)	9 (64.2)	1.06 (0.69–1.63)	
Alcohol use				0.892
NO	33 (39.3)	51 (60.7)	1	
YES	6 (37.5)	10 (62.5)	1.03 (0.68–1.56)	
Smoking habits				0.828
No	32 (39.5)	49 (60.5)	1	
Yes	7 (36.8)	12 (63.2)	1.04 (0.71–1.54)	

*p* values <0.05 were considered statistically significant. CI: confidence interval; PR: prevalence ratio.

**Table 2:** Distribution of patients with diabetes by comorbidity occurrence in relation to diabetic foot amputation.

Characteristics	Amputation		PR (95% CI)	*p*
	No *n* (prevalence %)	Yes *n* (prevalence %)		
Number of admissions for chronic complications^*∗*^ (*n* = 96)				**0.043**
1	18 (47.4)	20 (52.6)	1	
2 or 3	10 (25.0)	30 (75.0)	1.42 (1.00–2.03)	
>3	10 (55.6)	8 (44.4)	0.84 (0.46–1.54)	
Coronary insufficiency				0.096
No	26 (33.8)	51 (66.2)	1	
Yes	13 (56.5)	10 (43.5)	0.66 (0.40–1.08)	
Hypertension				0.152
No	8 (28.6)	20 (71.4)	1	
Yes	31 (43.1)	41 (56.9)	0.80 (0.58–1.09)	
Neuropathy				0.177
No	6 (66.7)	3 (33.3)	1	
Yes	33 (36.3)	58 (63.7)	1.91 (0.75–4.90)	
Vascular peripheral disease				0.179
No	11 (30.6)	25 (69.4)	1	
Yes	28 (43.7)	36 (56.3)	0.81 (0.60–1.10)	
Cerebrovascular insufficiency				0.380
No	31 (36.9)	53 (63.1)	1	
Yes	8 (50.0)	8 (50.0)	0.79 (0.47–1.33)	
Dyslipidemia				0.679
No	24 (40.7)	35 (59.3)	1	
Yes	15 (36.6)	26 (63.4)	1.07 (0.78–1.47)	
Nephropathy				0.697
No	28 (37.8)	46 (62.2)	1	
Yes	11 (42.3)	15 (57.7)	0.93 (0.64–1.35)	
Retinopathy				0.875
No	23 (39.7)	35 (60.4)	1	
Yes	16 (38.1)	26 (61.9)	1.03 (0.75–1.41)	

*p* values <0.05 were considered statistically significant (indicated in bold). CI: confidence interval; PR: prevalence ratio.

^*∗*^Chronic complications (coronary insufficiency, hypertension, and vascular peripheral disease).

**Table 3:** Distribution of patients with diabetes by age, time to diagnosis, and diabetic care in relation to foot amputation.

Characteristics	Amputation		PR (95% CI)	*p*
	No *n* (prevalence %)	Yes *n* (prevalence %)		
More than 3 appointments in the past year				**0.006**
No	7 (21.2)	26 (78.8)	1	
Yes	32 (47.8)	35 (52.2)	0.66 (0.50–0.89)	
Morisky test				**0.012**
2, 3, or 4	33 (45.8)	39 (54.2)	1	
0 or 1	6 (21.4)	22 (78.6)	1.45 (1.09–1.94)	
Age at diagnosis of diabetes				**0.030**
<40	5 (20.0)	20 (80.0)	1	
40 to 59	23 (42.6)	31 (57.4)	0.72 (0.53–0.97)	
≥60	11 (52.4)	10 (47.6)	0.60 (0.36–0.97)	
Diabetes monitoring				0.064
No	5 (23.8)	16 (76.2)	1	
Yes	34 (43.0)	45 (57.0)	0.75 (0.55–1.02)	
Glucose testing in the past year				0.073
No	3 (21.4)	11 (78.6)	1	
Yes	36 (41.9)	50 (58.1)	0.74 (0.53–1.03)	
Medical appointment in the past year				0.406
No	7 (31.8)	15 (68.2)	1	
Yes	32 (41.0)	46 (59.0)	0.86 (0.61–1.22)	
Annual medical appointment after diagnosis				0.462
No	9 (33.3)	18 (66.7)	1	
Yes	30 (41.1)	43 (58.9)	0.88 (0.64–1.23)	
Time since diagnosis (years)				0.586
<15	22 (41.5)	31 (58.5)	1	
≥15	17 (36.2)	30 (63.8)	1.09 (0.80–1.49)	

*p* values <0.05 were considered statistically significant (indicated in bold). CI: confidence interval; PR: prevalence ratio.

**Table 4:** Distribution of patients with diabetes by disease characteristics at admission in relation to foot amputation.

Characteristics	Amputation		PR (95% CI)	*p*
	No *n* (prevalence %)	Yes *n* (prevalence %)		
Previous conservative procedure				**<0.001**
No	8 (14.6)	47 (85.5)	1	
Yes	31 (68.9)	14 (31.1)	0.36 (0.23–0.57)	
Osteomyelitis				**<0.001**
No	32 (66.7)	16 (33.3)	1	
Yes	7 (13.5)	45 (86.5)	2.60 (1.71–3.94)	
Wagner criteria				0.051
2 or 3	16 (64.0)	9 (36.0)	1	
4	23 (30.7)	52 (69.3)	1.93 (0.95–3.91)	
Previous amputation				0.255
No	29 (42.6)	39 (57.4)	1	
Yes	10 (31.2)	22 (68.8)	1.20 (0.88–1.64)	
Diabetic foot characteristics				0.256
Neuropathic	1 (25.0)	3 (75.0)	1	
Ischemic	7 (70.0)	3 (30.0)	0.40 (0.13–1.21)	
Neuroischemic	31 (36.1)	55 (63.9)	0.85 (0.47–1.54)	
Age at admission				0.321
<60	13 (30.9)	29 (69.1)	1	
60 to 69	10 (40.0)	15 (60.0)	0.87 (0.59–1.27)	
≥70	16 (48.5)	17 (51.5)	0.75 (0.51–1.10)	
Glucose level at admission (mmol/L) (*n* = 98)				0.480
<7.77	8 (47.1)	9 (52.9)	1	
≥7.77	30 (37.0)	51 (63.0)	1.19 (0.74–1.92)	
Involvement of the other lower limb				0.701
No	27 (40.3)	40 (59.7)	1	
Yes	12 (36.4)	21 (63.6)	1.07 (0.77–1.48)	

*p* values <0.05 were considered statistically significant (indicated in bold). CI: confidence interval; PR: prevalence ratio; PR_adj_: adjusted prevalence ratio.

**Table 5:** Morisky test. Estimate of the prevalence ratio of the outcome to foot amputation in patients with diabetes using the Cox multiple regression model.

Characteristics	PR	PR_adj_ (95% CI)	*p*
Previous conservative procedure			**<0.001**
No	1	1	
Yes	0.36	0.37 (0.24–0.59)	
Previous use of antibiotics			**0.026**
No	1	1	
Yes	1.45	1.42 (1.04–1.92)	
Morisky test			**0.057**
2, 3, or 4 (compliance)	1	1	
0 or 1 (noncompliance)	1.45	1.26 (0.99–1.59)	

Statistically significant *p* values are indicated in bold. CI: confidence interval; PR: prevalence ratio; PR_adj_: adjusted prevalence ratio.

**Table 6:** Wagner criteria. Estimate of the prevalence ratio of the outcome to foot amputation in patients with diabetes using the Cox multiple regression model.

Characteristics	PR	PR_adj_ (95% CI)	*p*
Previous conservative procedure			**<0.001**
No	1	1	
Yes	0.36	0.39 (0.25–0.61)	
Previous use of antibiotics			**0.042**
No	1	1	
Yes	1.45	1.36 (1.01–1.82)	
Wagner criteria	1.97	1.65 (1.09–2.50)	**0.018**

*p* values <0.05 were considered statistically significant (indicated in bold). CI: confidence interval; PR: prevalence ratio; PR_adj_: adjusted prevalence ratio.
